# Prognostic value of serum creatine level in patients with vulvar cancer

**DOI:** 10.1038/s41598-019-47560-3

**Published:** 2019-07-31

**Authors:** Richard Schwameis, Magdalena Postl, Christine Bekos, Lukas Hefler, Alexander Reinthaller, Veronika Seebacher, Christoph Grimm, Stephan Polterauer, Samir Helmy-Bader

**Affiliations:** 1 Gynecologic Cancer Unit, Comprehensive Cancer Center, Medical University of Vienna, Waehringer Guertel 18-20, 1090 Vienna, Austria; 2 Department of Gynecology, Ordensklinikum Linz, Seilerstätte 4, 4020 Linz, Austria; 3 Karl Landsteiner Institute for Gynecologic Surgery and Oncology, Seilerstaette 4, 4020 Linz, Austria; 4 Karl Landsteiner Institute for General Gynecology and Experimental Gynecologic Oncology, Waehringer Guertel 18-20, 1090 Vienna, Austria

**Keywords:** Prognostic markers, Gynaecological cancer

## Abstract

Vulvar cancer is a rare malignancy with poor prognosis that generally occurs in elderly patients. The individual prognosis is difficult to assess. Serum creatinine levels are frequently elevated in elderly patients. Recent evidence have shown shown that - besides indicating kidney impairment - serum creatinine levels may be used to predict the survival in cancer patients. Several studies observed an association between elevated serum creatinine levels and poor prognosis in patients with solid tumors. In this retrospective cohort study, serum creatinine levels were evaluated in 170 patients with invasive vulvar cancer. Serum creatinine levels were correlated to established clinicopathologic factors. Univariate and multivariate survival analysis were performed. Elevated serum creatinine levels (>1.2 mg/dl) were significantly associated with both poor disease specific and overall survival. Three year overall survival rates were 74.8% and 32.5% for patients with serum creatinine levels of ≤ and >1.2 mg/dl, respectively. In a multivariate survival model, serum creatinine levels were significantly associated with overall survival independent of tumor stage and patients’ age. In conclusion, pretherapeutic serum creatinine levels may be useful as an independent prognostic parameter in patients with vulvar cancer.

## Introduction

Vulvar cancer is a rare gynecological malignancy with a slightly rising incidence rate^1,2^. It is associated with poor survival when compared to other gynecological malignancies. Although dependent on the tumor stage, it is difficult to predict the individual outcome of patients with vulvar cancer, because the survival of patients within the distinct tumor stages may vary significantly^3–5^.

For an individual outcome prediction of patients with vulvar cancer, several factors were considered in the past. On one hand, pathological parameters such as tumor resection margin, lymph node involvement and lymph node ratio were evaluated^3,6,7^. On the other hand, clinical parameters were also shown to be associated with patient survival. Recent studies observed an association between inflammatory biomarkers including C-reactive protein, hypoalbuminemia and fibrinogen to poor survival in patients with vulvar cancer^8,9^.

Interestingly, in a variety of malignancies including patients with sarcoma and urothelial carcinoma, renal impairment delineated by high serum creatinine levels has been associated with poor survival^10,11^. The underlying mechanism for this association is poorly understood^12^.

Impaired renal function is more frequent in patients with >65 years than in younger patients^13^. Although the age of patients developing vulvar cancer has slightly decreased within the last decades, the median age at diagnosis still remains at 68 years^1^. Thus it seems reasonable to assess whether renal impairment reflected by elevated kidney function parameters have a prognostic value in patients suffering from vulvar cancer.

Hence this study was performed to assess whether pretherapeutic serum creatinine levels have a prognostic value in patients with invasive vulvar cancer and whether it might serve as a cheap, readily available prognostic parameter.

## Materials and Methods

### Patients

This study included a total of 341 consecutive patients diagnosed with vulvar cancer that were treated between 1993 and 2016 at the Comprehensive Cancer Center Vienna, Vienna. Patients’ clinical data was harvested from available tumor databases and by review of electronic charts. Tumor staging was performed according to the 2009 International Federation of Gynecology and Obstetrics (FIGO) classification system^14^.

Diagnosis of vulvar cancer was established histologically by biopsy. Tumor assessment was performed by clinical examination, and if advanced tumor stage was assumed magnetic resonance imaging (MRI) and computed tomography (CT) were performed.

Patients were treated according to local standards and international guidelines. In early stages of the disease treatment included radical surgery with or without lymph node assessment. Clinically indicated patients received either adjuvant radiotherapy or in individual cases chemotherapy. In advanced stage disease palliative radiotherapy as well as chemotherapy with and without salvage surgery were performed. Table S1 of the supplemental material shows the treatment of patients in detail. All patients were included into the institutions follow up program that included clinical examination and imaging studies if necessary for a total of 10 years. All patients were seen four times annually for three years, twice per year in the following three years and, once annually for further four years. If recurrence was assumed biopsy was taken and imaging studies performed if required.

Approximately 24 to 72 hours prior to treatment patients were subjected to physical examination by a specialist for internal medicine. The examination included evaluation of medical history, a physical examination including blood pressure measurement, auscultation of the lungs, and evaluation of blood values including serum electrolytes, coagulation parameters, blood cell count liver function and renal function tests. If necessary additional examinations for example ECG, chest x-ray or pulmonary function tests were performed.

Patients with FIGO stage Ia and previously known chronic renal disease and patients where necessary values were missing were excluded from analysis.

### Ethics

Prior to initiation, this study was approved by the Institutional Review Board of the Medical University of Vienna (IRB approval number: 1901/2017). All patients gave consent to treatment according to institutional guidelines and to anonymized assessment of clinical data and treatment outcome. This was a retrospective trial. Therefore, the Institutional Review Board of the Medical University of Vienna waived the requirement to obtain distinct written informed consent from the patients.

Patient data were anonymized and de-identified prior to analysis. The study was performed according to the declaration of Helsinki, the ICH Harmonized Tripartite Guideline for Good Clinical Practice and the guidelines of the Institutional Review Board of the Medical University of Vienna.

### Creatinine measurements

As a part of the physical examination prior to treatment serum creatinine levels were assessed. The Creatinine Jaffé Gen. 2 test (COBAS CREJ2; Roche Diagnostics, Indianapolis, Indiana, USA) was used for serum creatinine level measurements and serum creatinine concentrations between 0.50 and 1.2 mg/dl were considered normal.

### Statistical analysis

All data is given as mean (±SD). Students’ t- test and one-way Anova were used to compare mean serum creatinine levels and clinic-pathologic variables, where appropriate. The product-limit method of Kaplan and Meier was used to calculate survival probabilities. Differences between groups were assessed by log-ranks test. Univariate and multivariate survival analysis were performed including patients’ age (≤ vs. >67.7 years) FIGO tumor stage (FIGO I vs. II vs. III vs. IV), histological grade (G1 vs. G2-G3), lymph node involvement (no involvement [N0] vs. involvement [N1]) and serum creatinine level. The results were analyzed for the endpoint of disease specific and overall survival, with overall survival being defined the primary endpoint. Overall survival was defined as time from the date of diagnosis to the date of death or date of last observation. Survivors were censored on the last date they were known to be alive. Disease specific survival was defined as time from date of diagnosis until date of death due to vulvar cancer (or sequels of vulvar cancer). Patients that died from causes other than vulvar cancer, and survivors were censored at the last date they were known to be alive. Multivariate cox regression models were established to assess serum creatinine levels as prognostic parameter for overall survival and disease specific survival. Serum creatine values were dichotomized at the cut off value of 1.2 mg/dl, since serum creatinine levels above 1.2 mg/dl are defined pathological. Variables that were significant prognostic parameters in univariate analysis were included into multivariate analyses, except for nodal status, which is an intrinsic part of FIGO stage. p-Values of ≤ 0.01 and ≤ 0.05 for univariate and multivariate analysis respectively were considered statistically significant. Hazard ratios (HR) and 95% confidence intervals (CI) are provided. Statistical analysis was performed using the Statistical Package for the Social Sciences statistical software (SPSS 24.0 for MAC, IBMCorp., Armonk, NY, USA). All data are available as Supplementary Database 1.

## Results

The data of 341 patients with vulvar cancer was available. A total of 171 patients had to be excluded from analysis because of either non-invasive vulvar cancer or incomplete clinical or laboratory data. In total, the data of 170 patients with invasive vulvar cancer (FIGO Ib-FIGO IVb) and complete clinical information was included in the analysis. Table 1 shows patient characteristics. In general, the mean serum creatinine level (SD) of patients with vulvar cancer was 0.91 ± 0.28 mg/dl. Pathologically elevated serum creatinine levels (≥1.20 mg/dl) were measured in 17 patients (10.0%). Table 2 shows the correlation of serum creatinine levels with clinicopathologic findings. Serum creatinine levels had no effect on patients’ treatment (Table S2 of the supplemental material). Elevated serum creatinine levels were associated with patient’s age (p < 0.001), patient’s performance status (p < 0.001) and histologic grading (p = 0.02).

**Table 1:** Study patient characteristics.

Parameter	N (%) or mean (SD)
No. of patients	170
Mean creatinine value	0.91 (0.28)
Patients’ age	67.7 (14.0)
FIGO Tumor stage	
FIGO I	95 (55.9)
FIGO II	30 (17.6)
FIGO III	32 (18.8)
FIGO IV	13 (7.6)
Histologic Grading	
G1	42 (24.7)
G2	97 (57.1)
G3	29 (17.1)
unknown	2 (1.2)
Nodal involvement	
N0	90 (52.9)
N1	56 (32.9)
Not assessed	20 (11.8)
unknown	4 (2.4)
ECOG status	
0	92 (54.1)
1	48 (28.2)
2	13 (7.6)
3	5 (2.9)
unknown	12 (7.1)
BMI	28.0 (6.9)
Metastatic disease	
M0	163 (95.9)
M1	7 (4.1)
Time of follow-up (months)	26.5 (IQR 10–62)*
Status at last observation	
Alive with no evidence of disease	94 (55.3)
Alive with stable disease	4 (2.4)
Progression	13 (7.6)
Deceased	59 (34.7)

*Given as median and interquartile range.

FIGO International Federation of Gynecologists and Obstetrics, ECOG European cooperative oncology group.

**Table 2:** Mean (SD) pre-treatment serum creatinine levels in patients with vulva cancer categorized by clinico-pathologic findings.

Parameter	Mean Creatinine (mg/dl)	p-value	Missing values
Age		<0.001^a^	0
≤67.7	0.79 (0.16)		
>67.7	0.99 (0.33)		
Tumor stage		0.49^b^	0
FIGO I	0.90 (0.28)		
FIGO II	0.97 (0.34)		
FIGO III	0.87 (0.20)		
FIGO IV	0.86 (0.36)		
Grading		0.02^a^	2
G1	0.73 (0.44)		
G2-3	0.88 (0.32)		
Nodal involvement		0.44^a^	4
N0	0.89 (0.25)		
N1	0.93 (0.30)		
Performance Status			
0	0.88 (0.22)	<0.001^b^	12
1	0.86 (0.22)		
2	1.15 (0.43)		
3	1.35 (0.77)		

^a^students’ t-test, ^b^One-way Anova, FIGO International Federation of Gynecologists and Obstetrics, ECOG European cooperative oncology group.

The median overall survival and disease free survival rates were 26.5 (IQR 10–62) months and 17.0 (IQR 5–53) months, respectively. In univariate analysis serum creatinine levels were evaluated as a continuous variable as well as a dichotomous variable using a cut-off of 1.2 mg/dl. The 3-year overall survival rate for patients with a serum creatinine level ≤1.2 mg/dl was 74.8% compared to 32.5% in patients with a serum creatinine level above 1.2 mg/dl (p < 0.001). In addition to FIGO tumor stage, patient’s age, nodal involvement, and serum creatinine levels considered as continuous variable and dichotomous variable were significantly associated with poor overall survival (Table 3).

**Table 3:** Univariate and multivariate overall survival analysis in 170 patients with invasive vulvar cancer.

Parameter	univariate		multivariate			
	3-ys OS	p-value	Comparison	HR	95%-CI	p-value
Serum Creatinine		<0.001		2.6	1.3–5.3	0.006
≤1.20	74.8%					
>1.20	32.5%					
Serum Creatinine		0.01				
FIGO tumor stage		<0.001	I vs. II vs. III vs. IV	1.8	1.40–2.30	<0.001
FIGO I	82.1%					
FIGO II	59.9%					
FIGO III	50.4%					
FIGO IV	52.7%					
Age		<0.001	>vs. ≤ 66.5 years	2.9	1.6–5.2	<0.001
≤67.7	82.3%					
>67.7	59.9%					
Grading		0.64				
G1	78.2%					
G2/G3	67.5%					
Nodal involvement		<0.001				
N0	81.7%					
N1	51.7%					

FIGO International Federation of Gynecologists and Obstetrics, OS disease specific survival, HR hazard ratio, CI confidence interval.

Nodal involvement was not included into the multivariate model, since the nodal status is a crucial part of FIGO stage.

**Figure 1:**
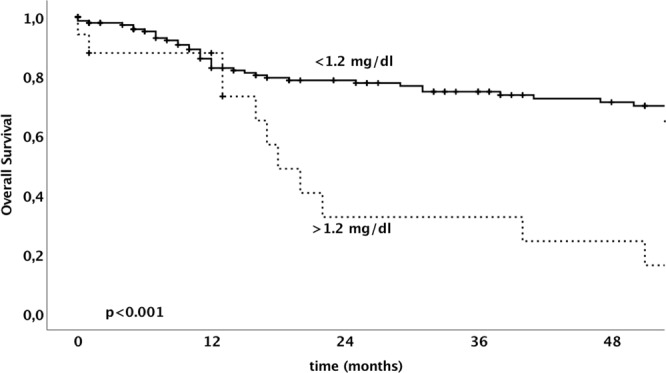
Kaplan–Meier analysis of overall survival of patients with vulvar cancer dependent on serum creatinine level using a cut-off of 1.2 mg/dI.

**Figure 2:**
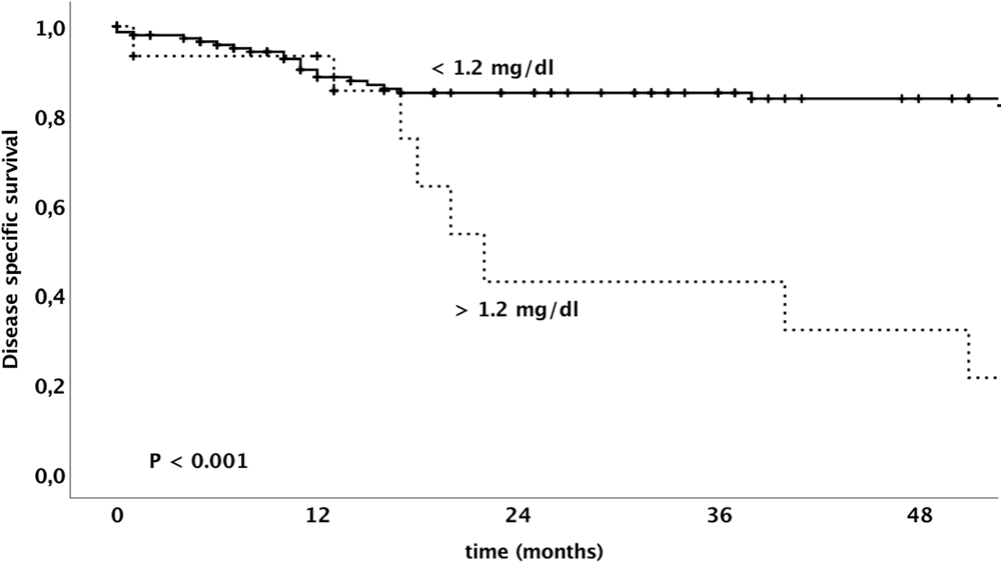
Kaplan–Meier analysis of disease specific of patients with vulvar cancer dependent on serum creatinine level using a cut-off of 1.2 mg/dI.

The overall survival and the disease specific survival of patients dependent on the serum creatinine level is shown in Figs 1 and 2, respectively. A multivariate analysis model revealed serum creatinine levels, FIGO tumor stage and patient’s age to be independently associated with overall survival in patients with vulvar cancer. Table 4 shows univariate and multivariate survival analysis for the endpoint of disease specific survival.

**Table 4:** Univariate and multivariate disease specific survival analysis in 170 patients with invasive vulvar cancer.

Parameter	univariate		multivariate			
	3-ys DSS	p-value	Comparison	HR	95%-CI	p-value
Serum Creatinine		<0.001	>vs. ≤ 1.2 mg/dl	3.8	1.6–9.0	0.002
≤1.20	85.1%					
>1.20	42.8%					
Serum Creatinine		0.005				
FIGO tumor stage		<0.001	I vs. II vs. III vs. IV	2.0	1.5–2.8	<0.001
FIGO I	93.6%					
FIGO II	59.9%					
FIGO III	63.1%					
FIGO IV	72.5%					
Age		0.014	>vs. ≤ 67.7 years	2.3	1.0–5.0	0.038
≤67.7	86.7%					
>67.7	76.5%					
Grading		0.07				
G1	89.2%					
G2/3	78.6%					
Nodal involvement		<0.001				
N0	92.1%					
N1	60.7%					

FIGO International Federation of Gynecologists and Obstetrics, DSS disease specific survival, HR hazard ratio, CI confidence interval.

Nodal involvement was not included into the multivariate model, since the nodal status is a crucial part of FIGO stage.

## Discussion

The present study investigated the prognostic value of pretherapeutic serum creatinine levels in patients with vulvar cancer. As a significant association between elevated pretherapeutic serum creatinine levels and poor overall survival in patients with invasive vulvar cancer, independent of established prognostic factors, was observed, several additional analysis were performed. Survival analyses for the endpoint of disease free survival were performed to support the finding of reduced overall survival. To elucidate the underlying mechanisms the relationship between serum creatinine levels and established risk factors were correlationally analysed.

The underlying mechanism of the association between elevated serum creatinine levels and diminished survival in patients with malignant diseases is subject to ongoing discussion. Several hypotheses have been proposed.

Firstly, elevated serum creatinine levels reflect impaired kidney function. Impaired kidney function is particularly known to increase cardiovascular mortality^15^. However, recent evidence showed that impaired kidney function is not only associated with cardiovascular mortality but also with non- cardiovascular mortality and with poor prognosis in different cancer patients^15,16^. Interestingly, the current study revealed that elevated serum creatinine levels were also associated with poor overall survival in patients with vulvar cancer. In addition, the disease specific survival of patients was also diminished in patients with elevated serum creatinine levels. This analysis was performed to corroborate the finding that elevated serum creatine levels are associated to poor overall survival. The observed association might indicate that this is not simply a side effect of the fact that patients with impaired kidney function have in general a decreased prognosis but may be potentially directly related to the malignant disease.

The creatine/creatinine metabolism not only plays a major role in energy production of muscle cells but has also been linked to carcinogenesis and cancer progression. Creatine and Phosphocreatine are essential energy sources donating ATP. Creatinine is a waste product of the creatine metabolism^17^. Preclinical studies showed during cancer progression creatine synthesizing enzymes to be significantly upregulated, while creatine levels are constantly decreasing^18^. Thus, it can be speculated that creatine is quickly utilized by the cancer cell as an energy resource, which may lead to creatinine as a waste product. Hence, it seems plausible, that elevated serum creatinine levels are associated with highly active tumors. In the current study, high creatinine levels were associated with grade 2 and 3 cancer. In addition, it seems therefore plausible that elevated serum creatinine levels are also associated with shortened disease specific survival in this study.

In contrast, serum creatinine levels were not associated with tumor stage. Thus, elevated serum creatinine levels were not simply caused by large tumor masses leading to urinary tract obstruction.

Secondly, evidence suggests that chronic low-grade inflammation may induce intra-renal vascular dysfunction that can lead to elevated serum creatinine levels^10^. Links between inflammation and cancer have been established for decades, and it has been shown that the tumor environment may sustain circle of tumor progression and inflammatory response^19,20^. Hence, elevated serum creatinine levels as seen in this study may mirror a chronic inflammatory reaction, triggered by the underlying malignancy.

In either case, if serum creatinine levels are increased in patients, a diagnostic algorithm as suggested by current guidelines should be applied to elucidate the underlying source of provenience^21^.

To our knowledge this is the first study to describe pretherapeutic elevated serum creatinine levels as an independent prognostic parameter for overall survival in patients with vulvar cancer. Undoubtedly, this study suffered from limitations due to its retrospective design. The treatment of patients with vulvar cancer changed and survival increased significantly over the last two decades. This might have had an impact on the results of the current study. On the other hand, we were able to include a relatively large homogenous cohort of 170 patients with vulvar cancer that was followed-up for a long period of time at a single institution.

Finally, we present pretherapeutic serum creatinine levels as a novel independent prognostic parameter in patients with vulvar cancer. This novel biomarker may be used for individual risk estimation, patient counseling and may be integrated in more advanced prognostic models for vulvar cancer.

In addition, it would be interesting to confirm our findings in a prospective study design and investigate whether pretherapeutic correction of high creatinine levels improves the survival in patients with vulvar cancer.

## Supplementary information

Supplemental Tables

Supplementary Database 1
