# Supplementary material for: Prognostic value of serum creatine level in patients with vulvar cancer
Source: Sci Rep. 2019 Jul 31;9:11129. doi: 10.1038/s41598-019-47560-3 (PMC6668438; doi:10.1038/s41598-019-47560-3)
Supplement: Supplementary file 1 — Supplemental Tables [file 41598_2019_47560_MOESM1_ESM.docx]

**Supplementary Information**

**Prognostic value of serum creatine level in patients with vulvar cancer**

Richard Schwameis^1^, Magdalena Postl^1^, Christine Bekos^1^, Lukas Hefler^2,3^, Alexander Reinthaller^1,4^, Veronika Seebacher^1^, Christoph Grimm*^1^, Stephan Polterauer^1,4^, Samir Helmy-Bader^1^

^1^ Gynecologic Cancer Unit, Comprehensive Cancer Center, Medical University of Vienna, Waehringer Guertel 18-20, 1090 Vienna, Austria

^2^ Department of Gynecology, Ordensklinikum Linz, Seilerstätte 4, 4020 Linz, Austria

^3^ Karl Landsteiner Institute for Gynecologic Surgery and Oncology, Seilerstaette 4, 4020 Linz, Austria

^4^ Karl Landsteiner Institute for General Gynecology and Experimental Gynecologic Oncology, Waehringer Guertel 18-20, 1090 Vienna, Austria

**Supplemental Table S 1**

Treatment of 170 patients with vulvar cancer according to FIGO stage.

| Stage/Treatment | Surgery | Surgery + Radiotherapy | Surgery + chemotherapy | Radiotherapy | Radiochemotherapy | Radiotherapy + salvage surgery | No therapy | Serum  creatinine (mg/dl) |
| --- | --- | --- | --- | --- | --- | --- | --- | --- |
| FIGO I | 89 (93.7) | 5  (5.3) | 0  (0.0) | 1  (1.1) | 0  (0.0) | 0  (0.0) | 0  (0.0) | 0.90 |
| FIGO II | 8  (26.7) | 19  (63.3) | 1  (3.3) | 0  (0.0) | 1  (3.3) | 1  (3.3) | 0  (0.0) | 0.97 |
| FIGO III | 2  (6.2) | 26  (81.3) | 0  (0.0) | 0  (0.0) | 4  (12.5) | 0  (0.0) | 0  (0.0) | 0.87 |
| FIGO IV | 2  (15.4) | 5  (38.5) | 0  (0.0) | 0  (0.0) | 2  (15.4) | 2  (15.4) | 2  (15.4) | 0.86 |

Additional Information:

FIGO III stage: One patient had solely surgery, because she had received inguinal radiation therapy prior the vulvar cancer due to lymphoma. Another one patient received only surgery, because final histology showed solely a micrometastasis (<2mm) In a single lymph node. Hence, no radiotherapy was suggested.

Figo IV stage: 2 patients received solely a surgical procedure. In one case patient died before start of adjuvant radiotherapy, in the other case patient had distant metastasis and refused adjuvant chemotherapy.

**Supplemental Table S 2**

Types of treatment of 170 patients with vulvar cancer in association with serum creatinine levels

| **Treatment** | **serum creatinine level (mg/dl)** |
| --- | --- |
| Surgery | 0.91 (0.29) |
| Surgery + radiotherapy | 0.93 (0.24) |
| Surgery + chemotherapy | 0.73 (0.0) |
| Radiotherapy | 1.3 (0.0) |
| Radiochemotherapy | 0.71 (0.26) |
| Radiotherapy + salvage surgery | 0.76 (0.29) |
| No Therapy | 0.85 (0.04) |

One-way ANOVA: p=0.08
